# Sensor-Agnostic, LSTM-Based Human Motion Prediction Using sEMG Data

**DOI:** 10.3390/s25175474

**Published:** 2025-09-03

**Authors:** Bon Ho Koo, Ho Chit Siu, Lonnie G. Petersen

**Affiliations:** 1 Department of Mechanical Engineering, Massachusetts Institute of Technology, Cambridge, MA 02139, USA; 2 MIT Lincoln Laboratory, Lexington, MA 02421, USA; 3 Department of Aeronautics and Astronautics, Massachusetts Institute of Technology, Cambridge, MA 02139, USA; 4 Institute for Medical Engineering and Science, Massachusetts Institute of Technology, Cambridge, MA 02139, USA

**Keywords:** motion prediction, deep learning, surface electromyography, LSTM

## Abstract

The use of surface electromyography (sEMG) for conventional motion classification and prediction has had limitations due to sensor hardware differences. With the popularization of deep learning-based approaches to the application of motion prediction, this study explores the effects that different hardware sensor platforms have on the performance of a deep learning neural network trained to predict the one-degree-of-freedom (DoF) angular trajectory of a human. Two different sEMG sensor platforms were used to collect raw data from subjects conducting exercises, which was used to train a neural network designed to predict the future angular trajectory of the arm. The results show that the raw data originating from different sensor hardware with different configurations (including the communication method, data acquisition unit (DAQ) usage, electrode configuration, buffering method, preprocessing method, and experimental variables like the sampling frequency) produced bi-LSTM networks that performed similarly. This points to the hardware-agnostic nature of such deep learning networks.

## 1. Introduction

Surface electromyography (sEMG) is a widely utilized non-invasive technique for capturing neuromuscular activity, offering valuable insights into human movement and control. In recent years, machine learning (ML) models have been increasingly employed to leverage sEMG signals for motion prediction, facilitating advancements in fields such as prosthetics, rehabilitation, and human–machine interaction [1]. However, a critical challenge in deploying such models lies in the variability introduced by different sEMG sensor hardware, including differences in electrode configuration, signal amplification, and noise characteristics. Understanding whether motion prediction models can generalize across different sensor types is essential for the development of robust and adaptable applications.

This study evaluates the performance of machine learning-based (bidirectional LSTM) motion prediction using two distinct sEMG sensor hardware systems. By training and testing predictive models on data acquired from both sensor types with differing physical as well as software parameters, we assess their relative performance in capturing movement-related neuromuscular patterns. By demonstrating comparable performance across data collected from distinct sEMG sensor hardware using identical data processing and network training methods, this study underscores the feasibility of sensor-agnostic ML approaches in motion prediction applications.

### 1.1. Hardware Dependence of EMG

The particular hardware form factor of an electromyography (EMG) sensor package is generally acknowledged to have a noticeable effect on the myoelectric signal collected. Broadly speaking, there are two ways in which the hardware can differ: in paradigm (such as surface or intramuscular) or in hardware configuration (such as due to manufacturing differences). Both of these vectors have been studied for their particular effects on collected data and the results for using different types. In general, a greater difference, both in signal and application performance, exists between surface and intramuscular EMGs [2], where they yield a statistically significant performance difference in favor of intramuscular EMGs, which show decreased classification errors. However, even among sEMG hardware, noticeable differences exist depending on the manufacturer, signal processing parameters innate to sensor packages, and other factors [3,4]. Nevertheless, the utilization of sEMG as a source for motion identification and the effects of set-up differences on the resulting performance has been observed in non-deep learning applications in the past [5].

### 1.2. Motion Prediction and Classification Using EMG

Surface electromyography (sEMG) is a non-invasive technique used to measure the electrical activity of muscles through electrodes placed on the skin. Unlike intramuscular EMG, which requires needle electrodes for direct muscle readings, sEMG is more practical for applications requiring continuous and non-disruptive monitoring of movement-related neuromuscular activity.

In motion prediction, sEMG is used to decode muscle activation patterns and anticipate voluntary movements before they physically occur [6,7]. Machine learning models analyze sEMG signal features—such as the amplitude, frequency content, and temporal variations—to establish relationships between neural drive and the resulting motion. This predictive capability is especially useful in prosthetic control, where sEMG-driven algorithms enable users to operate artificial limbs with intuitive muscle contractions [8]. It is also applied in rehabilitation, where movement intent can be inferred to assist patients with motor impairments, and in human–machine interfaces, where sEMG-based gesture recognition allows for hands-free control of external devices [9]. The effectiveness of sEMG-based motion prediction depends on the sensor quality, signal processing techniques, and robustness of machine learning models in handling variability across individuals, environments, and sensor types.

#### 1.2.1. Classification and Threshold-Based Algorithms

A number of studies utilize sEMG sensors as a data collection method for supervised learned—or otherwise threshold-based—motion classification. Often, sEMG data is collected through human subject exercises, and the raw data is processed through feature extraction and presented as training data for various algorithms. For example, k-nearest neighbor (KNN) approaches have been used to classify both hand and upper body motion with high accuracy [6,10,11]. In the context of the sEMG form factor, prior work suggests that certain paradigms or hardware packages perform better than others. Most importantly, the sensor has a statistically significant effect on the performance of these supervised and threshold-based classification algorithms [5].

#### 1.2.2. Deep Learning Methods

The body of work utilizing deep learning methods for motion prediction using EMG data is certainly rising as a topic of interest. Prior work suggests that deep learning-based motion prediction with sufficient accuracy is possible [6], especially in deep learning classifiers for motion trajectory and type prediction. Neural network methods have also been used for classification of prediction for human joint trajectories [10], dynamics (such as torques) [12], and the modes and classes of motion (such as between walking, running, or standing) [13], to give examples of the prior work estabilishing the possibility of utilizing deep learning methods for motion prediction using sEMG data. Generally, studies show that with sufficient EMG data quality and quantity, it is possible to train and deploy regression and classification networks that use data from EMG in their inference [14].

#### 1.2.3. Long Short-Term Memory Networks

A long short-term memory (LSTM) network is a type of recurrent neural network (RNN) specifically designed to model and learn from sequential data such as time series, speech, text, or biological signals. LSTM networks mitigate the vanishing gradient problem inherent in conventional RNNs through the introduction of a dedicated cell state and a system of gating mechanisms. In standard RNNs, repeated multiplication of gradients during backpropagation through time often results in exponential decay or growth, which severely limits the ability to capture long-term temporal dependencies. The LSTM architecture addresses this limitation by maintaining a cell state that provides a stable path for gradient flow across extended sequences. Information within the cell state is regulated by the forget, input, and output gates, which determine which past information is discarded, what new information is incorporated, and what information is propagated forward, respectively. This gating structure enables the selective retention and controlled update of information, thereby preserving error signals over long time horizons and preventing gradient vanishing. Consequently, LSTMs are able to learn both short- and long-range dependencies in sequential data with significantly greater robustness than traditional RNNs.

An extension of the LSTM architecture, bidirectional LSTM (BiLSTM), further enhances sequence modeling by introducing two parallel recurrent layers that process the input sequence in opposite directions: one forward in time and one backward. The outputs of these layers are typically concatenated, allowing the model to incorporate both past and future context when making predictions. This bidirectional structure is particularly advantageous in tasks where the entire input sequence is available, such as speech recognition, natural language processing, and biomedical signal analysis, as it enables the network to exploit information from both preceding and succeeding time steps to improve representational capacity.

### 1.3. EMG Hardware Dependence of Deep Learning and Regression

As discussed in Section 1.1, prior work shows that the hardware of the EMG sensor, whether through different paradigms or specifications, has an effect on the performance of both supervised learning and threshold-based classification algorithms. However, such a comparison has not been explicitly shown in the context of deep learning and regression prediction tasks. Based on the operating principles of each, we hypothesize that deep learning and regression algorithms may perform better than the classification algorithms mentioned prior.

We outline the methods and results of an experiment comparing the performance of a deep learning regression algorithm designed to predict the one-degree-of-freedom (DoF) angular motion trajectory about a human elbow using data from two different examples of sEMG sensor hardware from separate manufacturers.

## 2. Methods

### 2.1. sEMG Hardware

The two sEMG systems used were chosen due to their drastically different form factors and price points. The first system used was the Delsys (Natick, MA, USA) Trigno system. The second was 4 channels of the Advancer Technologies LLC (Raleigh, NC, USA) Myoware 2.0 system. See Table 1 for a list of notable differences between the two systems used in this study.

### 2.2. Experiment

We recruited five able-bodied subjects (4 male, 1 female; ages 22–30) with no current or historical upper-body musculoskeletal conditions for human subject testing. All recruitment and testing procedures were conducted in accordance with institutional review board-approved protocols. Recruitment was conducted without consideration of the fitness level, and no preparatory instructions were given prior to experimentation.

**Figure A1:**
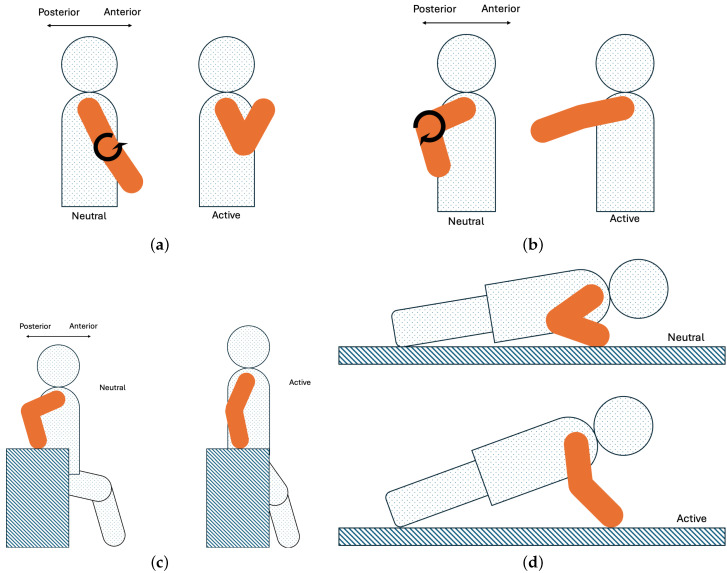
Series of diagrams representing the exercises prescribed to subjects. The exact magnitude depended on the range of motion of the subject. Exercises with weighted variants were identical to those with unweighted variants in terms of posture, with the addition of a preferred dumbbell as a mass. (**a**) Visual representation of a subject performing the elbow flexion motion from a lateral view. (**b**) Visual representation of a subject performing the elbow extension motion from a lateral view. (**c**) Visual representation of a subject performing the bodyweight dip motion from a lateral view. (**d**) Visual representation of a subject performing the push-up motion from a lateral view.

Subjects were instructed to perform three sets of 10 repetitions for each of the following upper-body exercises: unweighted elbow flexions (bicep curls), weighted elbow flexions, body weight dips, body weight push-ups, unweighted elbow extensions (tricep extensions), and weighted elbow extensions. The exercises are illustrated in Figure A1. While no explicit rest periods were prescribed between exercises, subjects were encouraged to rest as needed to avoid noticeable muscle fatigue. For the weighted exercises, participants selected a weight that could be lifted for a total of 30 repetitions while maintaining a consistent level of exertion throughout.

Surface EMG (sEMG) probes were placed on the biceps brachii, triceps brachii, brachialis, and brachioradialis, resulting in four sEMG channels. A parallel but separate Delsys goniometer sensor was also used during experimentation for ground truth data collection. Although placement was supervised, subjects were responsible for attaching the probes themselves, guided by a set of visual and photographic instructions. The placements were then checked by the research team for correctness, orientation, and consistency in accordance with the Surface ElectroMyoGraphy for the Non-Invasive Assessment of Muscles (SENIAM) project. If the placement did not appear to meet the required standards, then the subjects were asked to reapply the probes. The skin at each site was cleaned with isopropyl alcohol to improve both adhesion and signal quality. While the experimental environment was not specially engineered for electromagnetic interference reduction, it was selected after a preliminary evaluation to identify the area with the lowest baseline electrical noise. All sEMG and goniometer channels were sampled at a rate of 1 kHz. This resulted in the raw data collected to be approximately 2–3 million samples per channel per data collection session.

The order of exercises, experimental conditions, and mass presence was randomized per subject per trial. The specific permutation order was known to each subject beforehand and was confirmed to be unique across different subjects’ trials.

Due to the requirement of evaluating different sensor hardware, subjects conducted an additional session with an alternate sensor system, following an identical experimental set-up per subject and targeting the same muscles. A rest period was provided between the two sensor trials to prevent fatigue-related data contamination. The order of sensor use was randomized across the subjects; two subjects completed the Trigno-based session first, while the remaining three began with the Myoware 2.0 sensor.

The types of exercises, locations of the sEMG sensors, and protocol were selected in order to keep a consistent experimental protocol and data continuity from prior work, and thus the justification for selection was consistent as well. The exercises and observed muscles utilized in this study were selected in tandem due to their large and relatively isolated recruitment of said muscles in the particular exercise motions present in the study. Furthermore, the muscle’s large and relatively isolated nature makes signal integrity relatively robust [14].

### 2.3. Deep Learning Network Design

**Figure 1:**
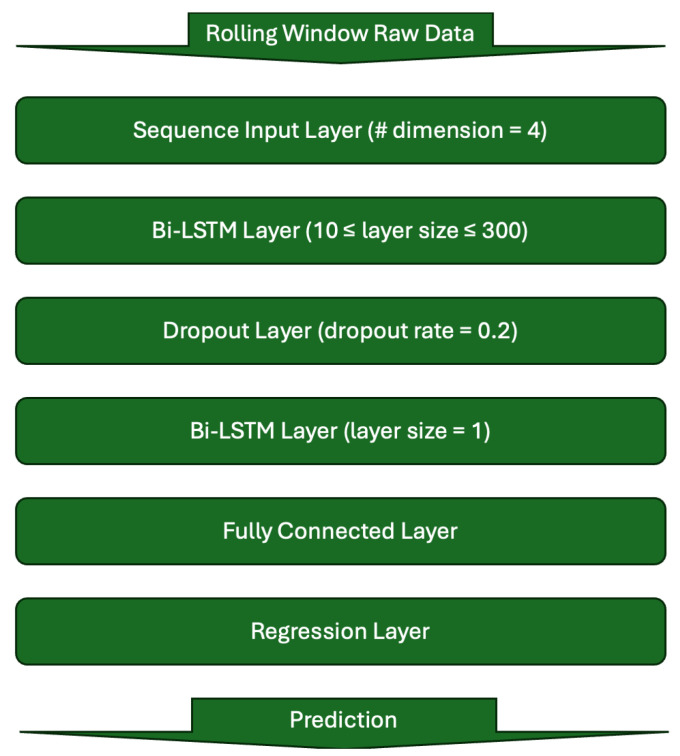
A visual representation of the LSTM network structure. Note that the layer size was one of the parameters swept for the performance comparison between the two sensor packages.

The network developed for sEMG-based motion prediction primarily consists of a bi-directional, multi-layer LSTM architecture. Its exact structure was adopted from prior work which conducted output error minimization optimization processes to identify the best performing network structure for the given task of predicting the regression output arm angle using sEMG inputs [14]. In general, Figure 1 visually shows the structure of the bidirectional LSTM networks evaluated and used in this study.

The cost function was a simple regression function, computing the mean squared error loss:

(1) $$loss=\sum_{i=1}^{R}{\left(t_{i}-y_{i}\right)}^{2}$$

where *R* is the number of responses, $t_{i}$ is the target output, and $y_{i}$ is the network’s prediction for the response *i*.

### 2.4. Computing Hardware

The inference presented in this study was conducted using the same local hardware and software combination. The hardware included an Intel i9-10900 CPU, an NVIDIA RTX 2080 Super 8 GB GPU, and 16 GB of RAM. The software environment included MATLAB 2022b running on the Windows 11 Pro operating system. Training of the network was performed on a high-performance computing unit at the MIT SuperCloud of the Lincoln Laboratory Supercomputing Center (Lexington, MA, USA) [15].

## 3. Results

Overall, the experimental results show that in the particular configuration presented in this study, there was no significant difference in performance between the networks trained on data gathered in the two different hardware platforms discussed.

There are three primary variables that have been shown [14] to affect to the performance of the network:

- Rolling window size: This is the size of the input rolling window, which corresponds to how much information is given per prediction about the current and past states of the target trajectory and the surrounding sEMG activation data (varied between 10 and 100 timesteps).
- Intersample distance: This is the temporal separation between the most up-to-date rolling window and the point for which the network is attempting to make a prediction. A large intersample distance means the network is attempting to make a prediction further into the future (varied between 10 and 300 timesteps).
- Layer size: This is the number of hidden units in the bi-LSTM layer of the network.

These variables are set when training, and each network is only capable of performing with the parameters it was trained for. The ranges of the above parameters were set to capture a large enough window of parameters to observe any potential trends that arose in the variation around its nominal value, which was observed in prior studies [14].

Varying the mentioned parameters yielded a total of 200 unique networks. We trained and evaluated these networks across both sensor hardware platforms described in Table 1. This approach enabled a comprehensive assessment of network performance under diverse parameter combinations. In training, a portion of the processed data was reserved as a validation set and used to assess the performance of each network. Specifically, 20% of all rolling windows generated using the raw data were reserved as a validation set.

**Figure 2:**
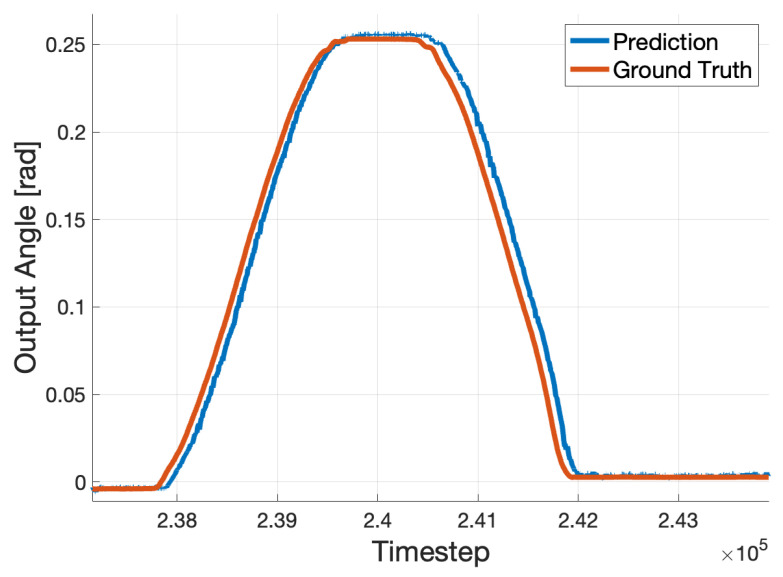
A visualization of a network’s prediction performance. Recall that predictions for each point in the trajectory were generated using raw sEMG data collected well in advance of each respective prediction without knowledge of the ground truth corresponding to each prediction.

Each network, in the regression output, produced a predicted trajectory. The difference between each prediction and the ground truth was defined as the error of each prediction. By using these errors across entire trajectories, it was possible to conduct statistical analysis. For visualization purposes, Figure 2 shows an example ground truth trajectory compared to a network’s prediction.

To compare the impact of hardware selection on model performance, we conducted paired *t*-tests for each parameter variation, isolating the effect of changing one parameter at a time. These statistical tests were performed to determine whether the observed differences in the error metrics between the two hardware platforms were significant. The results of these *t*-tests can be seen in Table 2. For each comparison, the other two parameters were held constant at the following values, which were within the selected parameter range: a window size of 10 timesteps, intersample distance of 10 timesteps, and layer size of 300 hidden units. Performance was evaluated based on a comparison of the mean error between the two hardware set-ups’ provided data generation results.

The results indicate that although slight variations in the mean error differences were observed, depending on the specific parameter and its value, the overall effect of the hardware platform on model performance was minimal. Across all tested configurations, the maximum difference in performance between the two platforms did not exceed approximately 1%.

## 4. Discussion

### 4.1. Benefits of Sensor-Agnostic sEMG Prediction

In this study, we show that the performance of an LSTM-based one-DoF angle prediction algorithm likely does not depend on the hardware platform used to collect the sEMG data, which is used to train and infer. This can be seen as a demonstration that motion prediction using deep learning, such as bidirectional LSTM networks, is sensor-agnostic. There are a number of points worth discussing regarding this conclusion.

The immediate effect is that future studies need not agree on a singular platform or singular method of data collection when attempting such motion prediction studies or applications. The sensor packages utilized in this study are as different as can be; apart from the unifying factor that they are both sEMGs, they are different in terms of communication method, software ecosystem, quality, configuration, capability, and even cost. It is reasonable to conclude that since they have demonstrated similar performance across a large range of parameters, it is possible that any reasonable sEMG system can as well. This, if true, means that future studies can incorporate sEMG sensor packages that best suit the other needs of the study without deep consideration into their effects on the results of the study.

An extended interpretation is that since it appears that LSTM-based motion prediction algorithms are robust to the input variability of training and inference data, it may also be possible that the input need not be sEMG sources at all. There is no innate setting defining the input to the algorithms explored in this study as sEMG. Therefore, it may be possible that entirely different input paradigms or combinations of paradigms can yield more desirable performance for whatever intended application with less consideration for sensor selection and system design on the input end. For example, a logical extension of an sEMG-based system would be invasive EMG or even an electroencephalogram (EEG)-based system or a hybrid system taking input from sEMG, EMG, and an EEG as a raw data source for training and prediction. If the difference between any of these is, from an algorithmic perspective, comparable to the difference between the two hardware platforms explored here, then they may perform similarly to the results shown in this study.

A practical extension of the results of this study is potentially greater flexibility in deep learning interpretations of sEMG data for a number of different applications. Conventionally, the uncertainty and, at times, demonstrated inconsistency of sEMG in analytical methods have deterred the use of sEMG as a sole data contributor in many applications in robotic control. With the results of this study, hardware variability can be mitigated as a potential source of variance in these applications through the use of deep learning data interpretation methods, leaving much of the optimization to the experimental set-up and environmental factor mitigation processes and approaches.

#### Ramifications of the Results

The results presented in this study have immediate and practical implementations. In future studies involving sEMG sensors, it can be advised that the selection, configuration, and deployment of the sensors should be guided more by the experiment and task at hand, rather than with the performance of the sensor as a focus. Furthermore, having shown that environmental factors (such as EMF inteference, subject-by-subject variability, the instantaneous physical state of subjects such as hydration, resting-state vitals, and problem application variability) conventionally considered highly sensitive do not have as great of an effect in performance when used for the purpose of training, an LSTM-based motion prediction algorithm, and subsequently predicting motion, studies can afford more leeway in exploring extreme cases for the environment, hardware, set-up, and task.

### 4.2. Limitations

There are certainly limitations to the both experimental methods and results presented in this study. First, while the hardware platforms were selected and configured to be as different as possible to cover the largest range in the claims of hardware agnostic performance, they are still two platforms. To more conclusively claim true hardware-agnostic performance in the context of deep learning motion prediction algorithms, a greater number of different sEMG systems should be explored in the future. Second, there are more environmental variables that affect the performance of the prediction network that was not explicitly tested in this study. For example, in the Methods section, we did not show the effects of fatigue, interfering multi-degree motion (such as stabilizing muscle activations), or physical preconditioning explicitly. While it is unlikely that these would have been kept consistent, the lack of instructions regarding these factors may have had an effect on the prediction algorithm’s training and inference. Furthermore, it can be argued that, while intentional in the context of this particular study, the uncontrolled parameters with regard to the particularities of sEMG (amplitude, noise, etc.) and their effects must be further studied. Finally, the subject sample size should be larger in order to prove the universality of the observations of this study. While due to the sampling rate, experiment length, and session count we were able to gather more than enough data to robustly train and confirm the performance of these networks, there is always the possibility of overfitting on an individual basis. Again, there was attempted mitigation of this effect through the use of data across multiple distinct sessions, but there may be underlying electro-myographic behaviors that are unique on an individual basis which may have served as an overfitting factor. Therefore, future studies should employ both more subjects and a larger number of sessions per subject.

## Figures and Tables

**Table 1:** A chart of notable differences between the Delsys Trigno and Advancer Technologies Myoware 2.0 sEMG systems.

	Delsys Trigno (Avanti Sensors)	Advancer Technologies Myoware 2.0
Communication	Wireless	Wired
Compound functionality	sEMG and IMU	sEMG only
DAQ	Proprietary	Arduino and Teensy ecosystem
Bandwidth	10∼850 Hz	20∼500 Hz
Innate buffering	∼32 ms per window of data	Real-time (no buffer)
Electrode configuration	Monopolar with reference node	Bipolar
Preprocessing	Instrument amplifier	Instrument amplifier and bandpass filter
Cost	∼USD 5000/channel	≤USD 200/channel

**Table 2:** Paired *t*-test comparisons across the two networks based on data from different sensor hardware set=ups with network parameters held constant, complete with standard error (SE), *p* value, and number of pairs compared per test (*N*).

Value	Δ Mean Error [rad]	SE	*p*	*N*
Comparison for window size				
10	0.0029	0.0009	0.0014	70
50	0.0003	0.0006	0.097	64
100	0.0046	0.0017	0.0092	34
Comparison for intersample distance				
10	0.0015	0.0006	0.0096	54
50	0.0028	0.001	0.0102	38
100	0.005	0.007	0.044	38
300	0.0044	0.002	0.053	38
Comparison for layer size				
10	0.0069	0.0019	0.018	20
50	0.0029	0.0011	0.0069	65
100	0.0002	0.0007	0.075	65
300	0.026	0.011	0.028	38

## Data Availability

The datasets presented in this article are not readily available because the data are part of an ongoing study. Requests to access the datasets should be directed to Bon H. Koo (bkoo1104@mit.edu) and Lonnie G. Petersen (lgpeters@mit.edu).

## Abbreviations

The following abbreviations are used in this manuscript:

(s)EMG	(Surface) electromyography
ML	Machine learning
DoF	Degree of freedom
(bi-)LSTM	(Bidirectional) long short-term memory
KNN	K-nearest neighbor
